# The heterogeneous treatment effect of adjuvant therapy with corticosteroids in patients with Community-Acquired Pneumonia: a review

**DOI:** 10.1186/s41479-026-00198-y

**Published:** 2026-04-25

**Authors:** Jim. M. Smit, A. Torres, P. A. van der Zee, Jesse H. Krijthe, Ignacio Martin-Loeches

**Affiliations:** 1 Data Science Group, Institute for Computing and Information Sciences, Radboud University, Nijmegen, The Netherlands; 2 Pattern Recognition & Bioinformatics Group, Delft University of Technology, Delft, The Netherlands; 3 School of Medicine, Institut D’Investigacions August Pi I Sunyer (IDIBAPS) Ciberes, University of Barcelona, Barcelona, Spain; 4 Department of Pulmonary Medicine, Erasmus MC - University Medical Center Rotterdam, Rotterdam, The Netherlands; 5 Department of Intensive Care Medicine, Multidisciplinary Intensive Care Research Organization, St James’ Hospital, Dublin, Ireland; 6 Department of Intensive Care, Erasmus MC – University Medical Center Rotterdam, Doctor Molewaterplein 40, Room Ne-411, Rotterdam, 3015 GD The Netherlands

## Abstract

Randomized Controlled Trials (RCTs) assessing adjuvant corticosteroids for hospitalized Community-Acquired Pneumonia (CAP) show mixed results, suggesting Heterogeneity of Treatment Effect (HTE). Current guidelines conflict: some restrict use to septic shock, while others advocate for broader application in severe CAP. Among three recent, large RCTs focused on severe CAP, however, only one demonstrated significant benefit, raising the question: should severity-based treatment guidance really be preferred?. The debate surrounding corticosteroid use in CAP is complicated by two issues: the lack of a unified definition for ‘severe CAP’, with definitions either based on the American Thoracic Society criteria or severity scores like the Pneumonia Severity Index, and the reliance on Aggregate Data Meta-Analyses (ADMAs). ADMAs synthesize trial results by categorizing entire RCTs as severe or non-severe, while most RCT populations included patients with varying disease severities. Individual Patient Data Meta-Analyses (IPDMAs) enable stratification on a patient-level. Our recent IPDMA utilized multivariate predictive HTE modelling in six RCT datasets to identify patient characteristics predicting corticosteroid benefit, which suggested C-reactive protein (CRP) as a predictor of benefit. Therefore, this finding was externally validated in two other RCTs, revealing statistically significant HTE found across baseline CRP subgroups (i.e., ≤ 204 mg/L vs. >204 mg/L), while no such HTE was found across severity-based subgroups. These findings may advocate for corticosteroid treatment guided by CRP instead of disease severity. CRP, however, is unlikely the sole driver of corticosteroids benefit, as other potential sources of HTE like imaging or cytokines (‘known unknowns’) or other unmeasured variables (‘ unknown unknowns’) may exist, and moreover, our IPDMA post-hoc suggested HTE across aetiology and treatment timing. Moving forward, the efficacy of precision medicine in CAP hinges on rigorous replication of HTE findings —being notoriously susceptible to false positives— in independent datasets, and harmonized and standardized data collection in future trials. Based on current evidence, CRP may serve as a pragmatic tool to guide corticosteroid treatment, but given to the drug’s “pendulum swings” from broad use to avoidance over the last decades, caution remains essential.

## Background

**Fig. 1:**
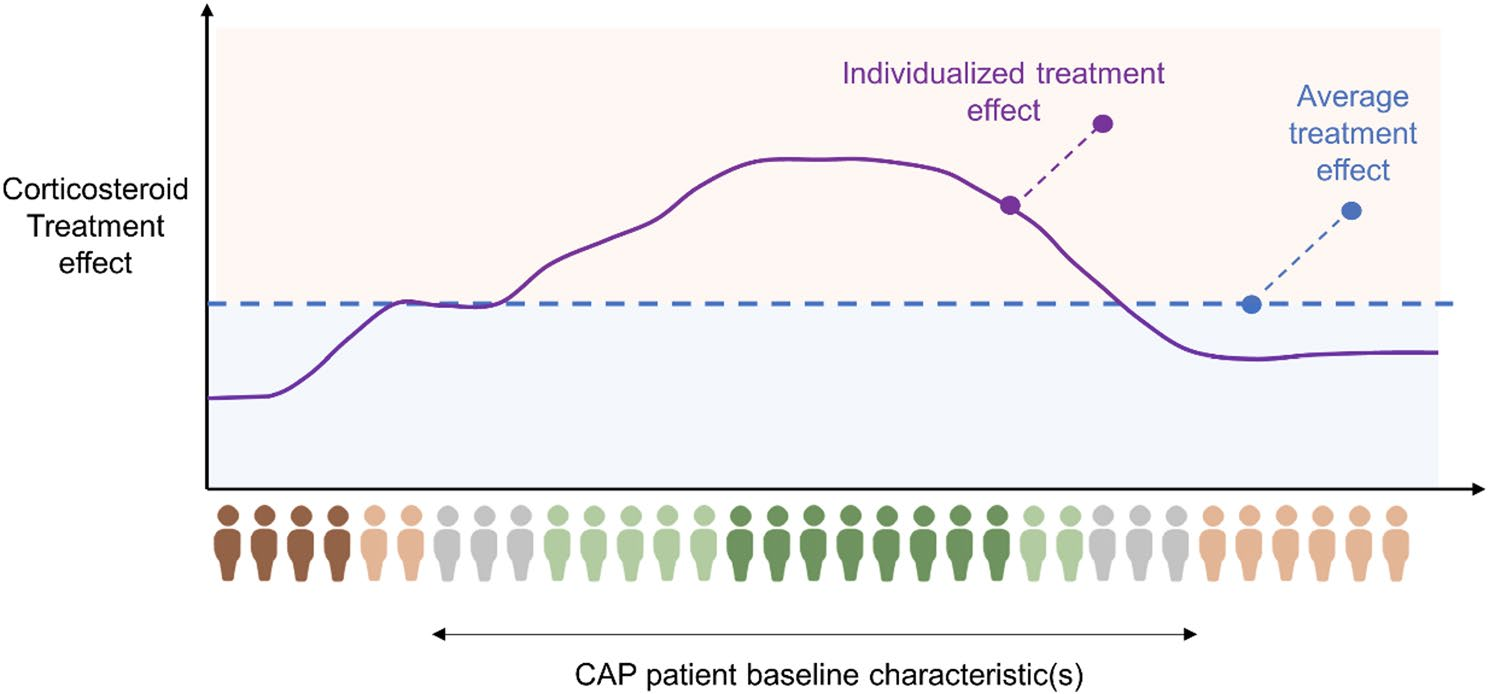
A visualization of heterogeneity of treatment effect (HTE). The effect on individual patients or subgroups can be larger, smaller or even in the opposite direction of the average treatment effect. This HTE can be driven by one or more patient characteristics. Figure inspired by Bica et al. [39]

Various randomized controlled trials (RCTs) comparing adjuvant therapy with corticosteroids with placebo in patients hospitalised with community-acquired pneumonia (CAP) showed mixed findings. A likely explanation is that only some patients with CAP benefit from corticosteroid treatment, while other do not benefit, or may even be harmed, a phenomenon known as *heterogeneity of treatment effect* (HTE; Fig. 1) [1, 2]. Despite evidence suggesting an average reduction in (short and long-term) mortality (Table 1), there is also evidence for adverse effects, including increased incidence of hyperglycaemia and hospital readmissions (Table 1) [3, 4]. Hence, rather than treating all hospitalized CAP patients, most current treatment guidelines favour corticosteroid treatment for CAP subgroups, in which the treatment benefits are expected to outweigh the risks. Which subgroups however, remains subject of debate.

Current guidelines offer conflicting recommendations. The 2023 European Respiratory Society, European Society of Intensive Care Medicine, European Society of Clinical Microbiology and Infectious Diseases, and Latin American Thoracic Association (ERS/ESICM/ESCMID/ ALAT) guideline [5] limits corticosteroid use to patients with septic shock, whereas the more recent Society of Critical Care Medicine (SCCM) and American Thoracic Society (ATS) guidelines [6, 7] advocate for a broader application, recommending corticosteroids for all patients with severe, bacterial CAP (including septic shock).

The 2025 REMAP-CAP trial [8], published after the completion of the evidence reviews conducted for the SCCM and ATS recommendations [6, 7], and exclusively focusing on severe CAP, however, reported no mortality benefit from adjuvant therapy with corticosteroids. This finding mirrors the 2022 ESCAPe trial [9], which also focused exclusively on severe CAP, and reported no benefit. Conversely, the 2023 CAPE-COD trial [10], again restricted to severe CAP, did demonstrate a significant benefit, and therefore acted as the main driver for the SCCM and ATS recommendations [6, 7]. Thus, among the three most recent RCTs [8–10], all of which exclusively enrolled severe CAP patients with substantial sample sizes, only one [10] reported a positive effect (Table 2). This raises a pertinent question: if corticosteroids truly benefit patients with severe CAP, why did two out of three rigorous trials fail to demonstrate this effect?.

## Main text

### ‘Severe’ CAP and the limitations of aggregate-data meta-analyses

The widely hypothesized HTE between severe and non-severe CAP patients is problematic for two main reasons: the absence of a unified definition for ‘severe’ CAP, and its primary support derived from inherently limited aggregate data meta-analyses (ADMAs).

The lack of a unified definition for severe CAP is reflected by the three most recent severe CAP RCTs [8–10], all three using different criteria to identify severe CAP. Likewise, in the ADMAs accompanying both the SCCM and ATS guidelines [6, 7], different criteria are used to define severe CAP. While the ATS use their own criteria [11] (mainly driven by requirement of invasive mechanical ventilation; IMV), the SCCM guidelines include criteria based on the Pneumonia Severity Index (PSI) [12], requirement of initial ICU admission, requirement of initial mechanical ventilation, or other severity scores like APACHE-II [13]. This mix of criteria leads to ambiguous recommendations for corticosteroid use, leaving clinicians with imprecise guidance.

**Fig. 2:**
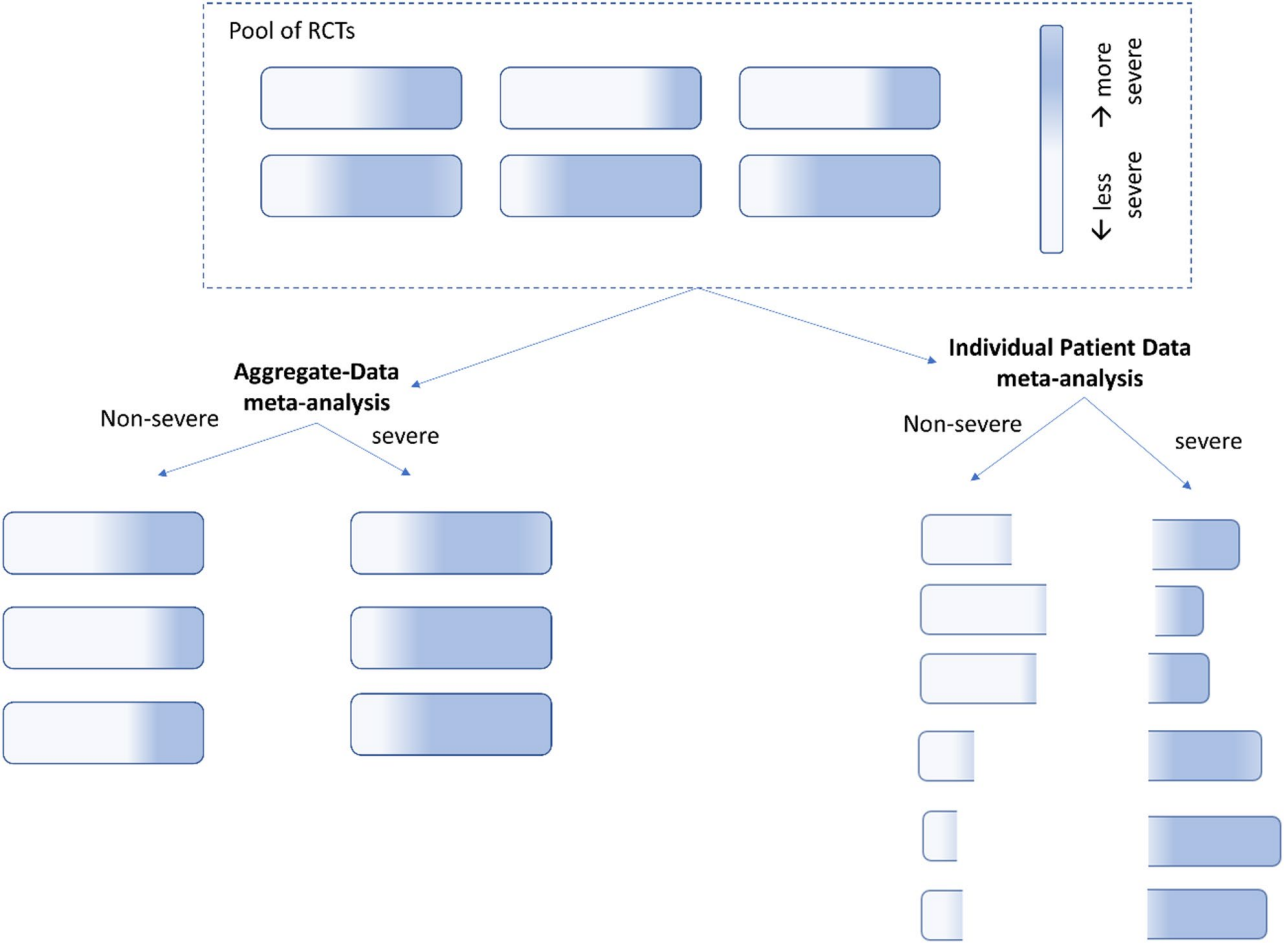
Schematic visualization of the limitations of an aggregate-data meta-analyse (AD-MA), in contrast to an individual patient data meta-analysis (IPDMA), for examination of heterogeneity of treatment effect across patient characteristic(s), such as disease severity

Moreover, even with a unified definition for severe CAP, HTE between non-severe and severe CAP cannot be confirmed through an ADMA, because this type of analysis relies on differences between rather than within RCTs [14]. ADMAs synthesize results from published studies by combining effect estimated found across individual trials. Hence, it is not possible to explore subgroup effects based on patient characteristics, if these characteristics also vary within trials. Likewise, the ADMAs supporting both SCCM’s and ATS’s guidelines [6] suffer from this limitation, categorizing entire RCTs as either ‘less severe’ or ‘severe’ CAP. In reality, all RCTs comparing adjuvant treatment with corticosteroids versus placebo enrolled patients with CAP across a spectrum of disease severities (Fig. 2). For instance, the studies by Blum et al. [15] and Meijvis et al., [16] categorized as ‘non-severe CAP’ by these AD-MAs, actually included patient populations where almost half (49% and 47%, respectively) presented with a PSI [12] Class IV or V. Both these studies reported null effects (with the Blum study [15] even trending toward harm), contributing to their conclusion that severe CAP patients benefit more; a conclusion biased by this limitation of whole-RCT categorization.

### Severity-based vs. CRP-based subgroups; evidence from individual patient data

Individual patient data meta-analyses (IPDMAs) overcome the limitations of ADMAs, enabling the investigation of HTE across subgroups by stratifying patients at an individual level (Fig. 2). For example, an IPDMA can re-analyze studies like those by Blum et al. [15] and Meijvis et al. [16] by splitting their populations into PSI-based non-severe and severe halves using any severity criteria available in these studies. Although it should always be preferred over an ADMA, IPDMAs are not immune to limitations. For instance, HTE findings in one RCT may not be *transportable* to another RCT due to differences in the patient population, randomized treatment regimes, or other trial aspects. Hence, decisions on which RCTs to include in an IPDMA should be taken with caution and based on domain knowledge.

To date, only two such IPDMAs on corticosteroid use in patients with CAP have been conducted [4, 17]. Our recent IPDMA [4], which included nearly all available RCTs predating the REMAP-CAP study [8], each with reasonably comparable inclusion criteria and corticosteroid regimes, extensively examined the HTE of corticosteroids through a multivariate, predictive HTE analysis. Here, rather than stratifying patients based on domain knowledge, a multivariate model ‘learns’ an effective stratification from data, a technique known as effect modelling [1, 18]. Based on data from six combined RCTs [15, 16, 19–22], encompassing twenty baseline variables (i.e., demographics, clinical parameters, laboratory values, and comorbidities), the multivariate model identified C-reactive protein (CRP) as the sole predictor of benefit from corticosteroids, with benefit predictions starting around CRP values of 200 mg/L and higher. Recognizing the susceptibility of data-driven approaches to overfitting, we subsequently validated the HTE across CRP-based subgroups in two other RCT datasets [9, 10] for which, crucially, data was received after the model and external validation plan were pre-registered [23]. Corticosteroids’ downregulating effect on proinflammatory cytokines can attenuate an excessive inflammatory response [24], which is a major driver of mortality in CAP [25]. As CRP synthesis is directly induced by pro-inflammatory cytokines, its serum concentration may serve as a surrogate marker for the magnitude of the inflammatory response and hence, a surrogate for the patient subgroup deriving the greatest benefit from corticosteroids.

Comparing HTE across CRP-based subgroups with HTE across CAP severity-based subgroups yielded clear results (Table 3): substantial and statistically significant HTE was observed across CRP-based subgroups, with greater benefit for those with higher baseline CRP. Conversely, no HTE was observed for PSI Class I-III vs. IV-V; in fact, odds ratio point estimates even suggested more benefit in the less severe group. Neither was increased benefit observed in severe versus non-severe CAP based on initial intensive care unit admission, or initial invasive mechanical ventilation. Significant HTE was observed between non-severe and severe CAP patients based on CURB-65 [26]. However, this was a post-hoc analysis; furthermore, the results contradicted the commonly hypothesised direction of effect by showing greater benefit in the non-severe group with CURB-65 scores 0–2 (Table 3).

These findings mostly align with the previous IPDMA by Briel et al., who analyzed five of the eight trials in our study (it predated the trials by Wittermans, Meduri, and Dequin), and did include the trial by Fernández-Serrano et al., which we excluded due to the high corticosteroid dose used.

Similar to our results, Briel et al. found no evidence of HTE between PSI classes I–III versus IV–V, or between patients immediately admitted to the ICU and those who were not. They also found no HTE across patients with or without ≥ 2 systemic inflammatory response syndrome (SIRS) criteria or prior antibiotic treatment, neither of which we studied. However, in contrast to our findings, they did not find evidence of HTE across baseline CRP levels when patients were split at 188 mg/L. Our contrasting positive finding of HTE across baseline CRP levels (using a slightly different, data-driven threshold of 204 mg/L), hence, emerged with the addition of evidence from newer RCTs.

### CRP only?

As our findings only provided evidence of HTE across baseline CRP levels [4], these raise the question whether the benefit is truly solely driven by CRP. Given the complex pathophysiology and numerous factors in the inflammatory response, the answer, probably, is no.

A treatment strategy using baseline CRP as the only criterion could lead to treating patients who do not benefit or could be harmed. For example, our study [4] also showed a non-significant trend toward harm in patients with viral CAP, including influenza, regardless of baseline CRP. Point estimates even suggested a stronger trend of harm for patients with viral CAP without a bacterial co-infection. Although these findings are biologically plausible, and aligning with earlier observational studies [27, 28], these were based on post-hoc analyses.

In addition, using a CRP-only strategy may withhold corticosteroids from patients who may benefit. For instance, patients with recent symptom onset might still be in the phase where their CRP is rising and has not yet exceeded the 200 mg/L threshold at hospitalization, even though it would shortly after. Similarly, patients who received antibiotic treatment prior to hospitalization could have lower CRP values than they would have otherwise. A treatment strategy based only on baseline CRP would cause these patients to miss out on potentially beneficial treatment.

Also, CRP concentrations may be elevated for reasons other than CAP (such as chronic inflammatory conditions, cardiometabolic conditions, tissue damage, active neoplastic disease or lifestyle factors like obesity) [29–32], and therefore might be a non-specific marker. Such conditions, however, typically result in elevations well below the discussed threshold around 200 mg/L that may be useful for corticosteroid guidance.

Finally, our understanding of the HTE is limited by the available data. Despite the many factors measured in our IPDMA (demographics, clinical parameters, lab values, and comorbidities), other potential sources of HTE, such as radiological abnormalities, need for non-invasive ventilation, or cytokine data, were not sufficiently measured, while other potential HTE sources may not even be considered, leaving many ‘known unknowns’ and ‘unknown unknowns’. This highlights that our knowledge of the HTE of corticosteroid treatment currently is, and, to a certain extent, will always be, constrained by the data we have.

### Corticosteroid treatment protocols: drug, timing, dose and adaptive regimes

The RCTs included in our IPDMA showed variations in treatment arms, each with its unique combination of corticosteroid type, dose, duration, and timing. Therefore, even if a treatment strategy were guided by baseline CRP, clear guidance on how to deliver the treatment is still warranted. Post-hoc, we also investigated HTE across these treatment variations (Table 3).

The analysis, which converted all corticosteroids to equivalent hydrocortisone quantities, found no HTE based on cumulative dose by day seven. However, it did find significant HTE based on corticosteroid type, specifically showing a benefit for hydrocortisone versus other types when adjusting for baseline CRP levels (≤ 204 mg/L or > 204 mg/L; adding this as a binary variable to the regression model used for the interaction test). However, the negative results from the REMAP-CAP trial [8] (not included in our IPDMA), which used hydrocortisone, is conflicting evidence of the apparent superiority for hydrocortisone over other corticosteroid molecules.

Post-hoc, the CAPE-COD trial [10] provided data on treatment timing, showing that all benefits were confined to the subgroup treated within 24 h of hospital admission, reflected by significant HTE. Furthermore, CAPE-COD [10] uniquely used a response-dependent treatment regimen, choosing a total duration of 8 or 14 days based on pre-defined criteria: patient breathing spontaneously; PaO_2_/FiO_2_ > 200 mmHg; Sequential Organ Failure Assessment score increased with respect to day 1; and an estimation by the clinician in charge of successful discharge by day 14. Given this trial’s strong positive findings, such a response-dependent treatment regimen may be superior to fixed-duration regimens. Both these hypotheses regarding treatment timing and adaptive regimes, however, are solely based on the CAPE-COD trial [10]. 

### Multivariate HTE analyses

The earlier IPDMA by Briel et al., [17] employed a traditional subgroup analysis with pre-defined, ‘one-variable-at-a-time’ subgroups. This more traditional approach may suffer from limitations including low power and multiple comparisons, and depends on subjective human judgements. Furthermore, patients could belong to multiple overlapping subgroups that may experience treatment effects of varying size and direction. Predictive HTE approaches, as showcased in our study, aim to overcome some of these limitations, using multivariable models that enable analysing HTE across multiple patient characteristics simultaneously, selecting interaction(s) independent of subjective human judgements. For instance, whereas the CRP subgroup analysis performed in the IPDMA by Briel et al. [17] was chosen based on prior hypotheses, the selection of CRP subgroups in our IPDMA was purely data-driven. Paradoxically, however, our multivariate approach resulted in a model that selected only one variable: CRP. While the existence of other sources of HTE of corticosteroids beyond CRP is plausible, the challenge lies in identifying and, importantly, evaluating these.

### Subgroups or subphenotypes?

Subphenotyping is a multivariate strategy for exploring HTE which is especially popular in the ICU community [33]. The 2023 ESICM guidelines on acute respiratory distress syndrome [34] discuss the topic, defining a subgroup as a ‘subset of patients which may be defined using any cut-off in a variable,’ while a subphenotype as a ‘distinct subgroup that can be reliably discriminated from other subgroups based on a set or pattern of observable or measurable properties, typically based on a data-driven assessment of a multi- dimensional description of traits, and reproducible in different populations.’ Following these definitions, patients with an elevated baseline CRP could be considered both a subgroup (ie, based on a single variable and cut-off) and a subphenotype (because a data-driven, multivariate model identified this subgroup, and the HTE across these was reproduced in other populations). Rather than making a strict distinction, it may be more practical to call any patient selection (based on one or more characteristics) a subgroup, and focus on categorizing the different data-driven approaches used to identify HTE. Beyond sub-phenotyping, which uses (typically clustering or latent class) models trained only on patient covariates, other approaches could be grouped in roughly two categories: risk modeling and effect modeling [18, 35]. Risk modeling stratifies patients by predicted risk, using models that forecast outcomes based on covariates (for instance, examining HTE across PSI classes). Effect modeling incorporates treatment assignments during training to model treatment-covariate interactions and estimate individualized treatment effects. The model that selected CRP as the single variable is an example of this approach. While theoretically ideal for detecting HTE, effect modeling is also highly susceptible to overfitting. Regardless of the chosen strategy, rigorous evaluation of any HTE findings is essential before they are used to inform clinical practice [36]. 

### HTE evaluation: risky business

The statistical approach of evaluating HTE findings involves testing for a subgroup-treatment effect interaction. However, highly significant interactions can occur by chance. For instance, a post-hoc analysis of the European Carotid Surgery Trial found a significant variation in endarterectomy’s effect for severe carotid stenosis by month of birth (*P* < 0.001), a finding that would have been difficult to ignore if it had been in relation to another more plausible variable [36]. Similarly, the HTE observed between hypo- and hyperinflammatory sub-phenotypes for high versus low PEEP in the ALVEOLI trial [37] was not replicated in another trial [38], suggesting the initial finding, which spurred sub-phenotyping in critical care, may have been a false positive. Therefore, the best test of subgroup analysis validity is not significance, but *replication*: finding anticipated, statistically significant HTE in data which was unavailable at the moment the HTE analysis is pre-registered or pre-specified. To ensure HTE is anticipated, data ideally remains unseen until pre-registration of both the HTE model and evaluation protocol.

### Further research and a call for data harmonization

Returning to our introductory question; why did two [8, 9] out of three most recent trials fail to demonstrate effect? As all of these exclusively included patients with severe CAP (according to the ATS/IDSA definition [11]), it seems clear that disease severity alone cannot explain the divergent results. Differences in corticosteroid type, dosage, or timing are also unlikely drivers, as both CAPE-COD [10] (with positive findings) and REMAP-CAP [8] (with negative findings) used hydrocortisone with similar dosages, and initiated treatment within comparable time frames (Table 2). Instead, adaptive dosing (exclusive to CAPE-COD [10]), the inclusion of influenza patients (6–10% in eSCAPE [9] and REMAP-CAP [8], but excluded in CAPE-COD [10]), and variations in the baseline magnitude of inflammatory response quantified by CRP (median CRP level of 249 mg/L in CAPE-COD [10] compared to 106 mg/L in eSCAPE [9]) may play a role (Table 2).

As discussed previously, comparing differences between the overall populations of different trials is limited, as these characteristics may vary widely within trials as well (Fig. 2); studying HTE using individual patient data is preferred. In that light, the recent REMAP-CAP [8] trial offers a unique opportunity to further evaluate our HTE findings. Since our IPDMA analysis [4] predated REMAP-CAP [8], its data could serve as an independent set to further validate HTE across CRP subgroups (so-far not reported for REMAP-CAP [8]), as well as other post-hoc hypotheses, such as those related to treatment timing and disease aetiology. Statistically significant HTE across baseline CRP subgroups in the REMAP-CAP [8] dataset al.one should not be expected due to limited power. However, observing an HTE trend in the same direction (i.e., point estimates suggesting more benefit for patients with elevated baseline CRP) would reinforce, whereas similar point estimates or a trend in the opposite direction would weaken the evidence for HTE across baseline CRP levels.

Furthermore, both existing data as well as this new data could be further used to explore other multivariate HTE approaches, employing unexplored data-driven techniques. While this could lead to new insights, it remains crucial to replicate any new HTE findings in an independent dataset to ensure their validity.

New data types, such as additional inflammatory markers like cytokines, could also offer valuable insights. However, in past trials [15, 16, 22], inconsistent measurement techniques have limited the comparability of this cytokine data. This highlights a critical need for future trial datasets to collect clinical, radiological, and biological markers in a more harmonized, standardized way. Doing so would pave the way for a shared infrastructure for precision trials in CAP and other infectious diseases, making it easier to explore HTE sources using data-driven techniques.

## Conclusions

CAP is a heterogeneous disease, and RCTs studying the effect of corticosteroids yielded mixed results. While all current guideline recommend corticosteroids in presence of shock, only the two most recent guidelines broadened this, advocating corticosteroids treatment in severe CAP (with an exception of influenza CAP). Currently, however, the evidence of HTE for corticosteroids is stronger across baseline CRP levels than across disease severity. Although CRP unlikely offers a definitive biological explanation of the HTE of corticosteroids, it may serve as a pragmatic tool to guide corticosteroid therapy. Still, caution remains warranted: corticosteroids have been, and may remain a “pendulum drug,” swinging from broad use to avoidance over the last decades.

**Table 1:** Summary of key findings regarding average treatment effects of corticosteroids from our recent individual patient data meta-analysis [4]. IMV=invasive mechanical ventilation, †statistically significant effect, ‡in three of the four trials from which hyperglycaemia data was collected, no standardised definition was used

	Outcome	Average effect, Odd ratio, (95% CI)	Average effect, rate reduction (%, 95% CI)	Data in analysis	Summary
Primary endpoint	30-day mortality	0·72 (0·56 to 0·94)†	2.2% (0.6 to 3.7)	8 RCTs [9, 10, 15, 16, 19–22]; 3,224 patients	Significant benefit; based on** pre-registered analysis**
Secondary endpoints	90-day mortality	0·71 (0·51 to 0·99)†	2.8% (0.4 to 5.2)	4 RCTs [10, 15, 19, 21]; 1,745 patients	Significant benefit; based on **post-hoc** analysis
	28-day initiation of IMV	0·59 (0·42 to 0·82)†	4.8% (2.1 to 7.5)	4 RCTs [10, 15, 19, 21]; 1,568 patients	Significant benefit; based on **post-hoc** analysis
	28-day initiation of vasopressors	0·54 (0·40 to 0·72)†	6.9% (4.0 to 9.7)	3 RCTs [10, 15, 19]; 1,625 patients	Significant benefit; based on **post-hoc** analysis
	Hospital readmission	1·95 (1·24 to 3·07)†	-3.3% (-5.3 to -1.5)	4 RCTs [15, 16, 20, 22]; 1,633 patients	Significant **adverse** effect; based on **post-hoc** analysis
	Hospital length of stay	-	1.0† (0.5 to 1.0)	6 RCTs [15, 16, 19–22]; 1,831 patients	Significant benefit; based on **post-hoc** analysis
	ICU length of stay	-	2.0† (0.0 to 2.0)	4 RCTs [10, 15, 19, 21]; 930 patients	Significant benefit; based on **post-hoc** analysis
Adverse events	Hyperglycaemia‡	2·50 (1·63 to 3·83)†	-12.0% (-17.0 to -6.9)	4 RCTs [16, 20–22]; 683 patients 683 patients	Significant **adverse** effect; based on **post-hoc** analysis
	Hospital-acquired infection	0·88 (0·63 to 1·22)	0.9% (-1.6 to 3.9)	7 RCTs [10, 15, 16, 19–22]; 2,650 patients	No significant effect; based on **post-hoc** subgroup analysis
	Gastro-intestinal bleeding	0·93 (0·47 to 1·85)	0.1% (-0.8 to 1.0)	5 RCTs [10, 15, 19–21]; 1,958 patients	No significant effect; based on **post-hoc** analysis

**Table 2:** Summary of study findings and overall characteristics of the three most recent randomized trials that exclusively included patients with severe CAP; eSCAPE [9], CAPE-COD [10] and REMAP-CAP [8].

	eSCAPE (Meduri et al. [9])	CAPE-COD (Dequin et al. [10])	REMAP-CAP (Angus et al. [8])
Main finding (average treatment effect)	No significant effect of corticosteroids on mortality: 30-day mortality 13.7% in corticosteroid group vs. 14.4% in control group.	Significant effect of corticosteroids on mortality: 30-day mortality 6.8% in corticosteroid group vs. 12.4% in control group.	No significant effect of corticosteroids on mortality: 90-day mortality 15% in corticosteroid group vs. 9.8% in control group.
Participants with severe CAP (according to ATS/IDSA definition [11])	100%	100%	100%
Corticosteroid type	Methylprednisolone	Hydrocortisone	Hydrocortisone
Corticosteroid dose (cumulative dose on study day 7, in hydrocortisone equivalents, mg)	1600	1100 or 1500†	1400
Timing of Corticosteroid initiation	Not reported.	56.9% of participants within 24 h, and 88.8% participants within 48 h after hospital admission.	Median time between hospital admission to study enrolment between 0.7 and 0.8 days. The first dose of hydrocortisone was given before midnight of the first study day in 94.2% of participants.
Adaptive regime?	No	Yes†	No
Participants with influenza	6.6 to 7.3%	Excluded	8 to 10%
Baseline CRP (median, IQR)	106 mg/L (24 to 204.5)	249 mg/L (117 to 353)	Not reported.

†The, CAPE-COD [10] uniquely used a response-dependent treatment regimen, choosing a total duration of 8 or 14 days based on pre-defined criteria: patient breathing spontaneously; PaO2/FiO2 > 200 mmHg; Sequential Organ Failure Assessment score increased with respect to day 1; and an estimation by the clinician in charge of successful discharge by day 14

**Table 3:** Summary of key findings regarding heterogeneity of treatment effect (HTE) of corticosteroids on 30-day mortality, from our recent individual patient data meta-analysis [4]. IMV=invasive mechanical ventilation, †statistically significant HTE, ‡excluding negative findings of REMAP-CAP using hydrocortisone

Studied HTE Category	HTE Across …	Subgroups	Relative effects, Odd ratio, (95% CI)	Absolute effects, % 30-day mortality reduction, (95% CI)	Data in subgroup analysis	Summary
**Primary Analysis**	Baseline C-reactive protein (CRP)	≤ 204 mg/L vs. >204 mg/L	0·98 (0·63 to 1·50) vs. 0·43 (0·25 to 0·76)†	0.3 (-4.2 to 4.1) vs. 6.9 (2.8 to 10.4)	2 RCTs [9, 10]; 1,355 patients	**Significant** HTE, suggesting benefit for higher baseline CRP; based on **pre-registered** subgroup analysis
	Pneumonia severity index (PSI)	Class I-III vs. Class IV-V	0·60 (0·25 to 1·42) vs. 0·72 (0·54 to 0·95)	0.8 (-0.5 to 1.9) vs. 3.3 (1.1 to 5.5)	8 RCTs [9, 10, 15, 16, 19–22]; 3,224 patients	No significant HTE; based on **pre-registered** subgroup analysis
**Alternative Severity Criteria**	CURB-65	Score 0–2 vs. Score 3–5	0·53 (0·36 to 0·78) vs. 1·32 (0·60 to 2·89)†	3.1 (1.5 to 4.7) vs. -3.9 (-11.9 to 4.5)	6 RCTs [10, 15, 16, 20–22]; 2,315 patients	**Significant** HTE, suggesting more benefit in lower CURB-65 scores; based on **post-hoc** subgroup analysis
	Initial ICU admission	No vs. Yes	0·85 (0·52 to 1·37) vs. 0·50 (0·33 to 0·78)	0.6 (-1.0 to 2.2) vs. 6.1 (3.0 to 9.0)	6 RCTs [10, 15, 16, 19–22]; 2,663 patients	No significant HTE; based on **post-hoc** subgroup analysis
	Initial need for IMV	No vs. Yes	0·57 (0·37 to 0·88) vs. 0·63 (0·26 to 1·50)	3.4 (1.1 to 5.3) vs. 5.3 (-3.5 to 14.0)	4 RCTs [10, 16, 21, 22]; 1,619 patients	No significant HTE; based on **post-hoc** subgroup analysis
**Aetiology / Pathogen**	Non-COVID, viral CAP	No vs. Yes	0·57 (0·40 to 0·81) vs. 1·69 (0·58 to 4·88)	3.3 (1.6 to 5.0) vs. -2.6 (-7.1 to 1.5)	7 RCTs [10, 15, 16, 19–22]; 2,502 patients	No significant HTE; based on **post-hoc** subgroup analysis
	Non-COVID, viral CAP, without bacterial co-infection	No vs. Yes	0·58 (0·41 to 0·82) vs. 2·02 (0·59 to 6·92)	3.2 (1.5 to 4.8) vs. -4.0 (-9.4 to 1.0)	7 RCTs [10, 15, 16, 19–22]; 2,502 patients	No significant HTE; based on **post-hoc** subgroup analysis
	Influenza CAP	No vs. Yes	0·58 (0·40 to 0·84) vs. 1·61 (0·51 to 5·02)	2.9 (1.4 to 4.6) vs. -3.6 (-11.5 to 4.2)	7 RCTs [10, 15, 16, 19–22]; 2,382 patients	No significant HTE; based on **post-hoc** subgroup analysis
	Influenza CAP, without bacterial co-infection	No vs. Yes	0·59 (0·41 to 0·85) vs. 1·75 (0·47 to 6·57)	2.8 (1.3 to 4.5) vs. -4.4 (-13.4 to 4.2)	7 RCTs [10, 15, 16, 19–22]; 2,382 patients	No significant HTE; based on **post-hoc** subgroup analysis
**Corticosteroid type**,** dose**,** treatment timing**	Corticosteroid type‡	Hydrocortisone vs. others	0·43 (0·27 to 0·70) vs. 0·93 (0·68 to 1·29)†	7.2 (3.8 to 10.7) vs. 0.4 (-1.4 to 1.9)	8 RCTs [9, 10, 15, 16, 19–22]; 3,224 patients	**Significant** HTE; based on **post-hoc** subgroup analysis
	Study day 7 cumulative dose (hydrocortisone equivalent)	< 1,000 mg vs. 1000–1500 mg vs. > 1500 mg	0·80 (0·38 to 1·69) vs. 0·69 (0·47 to 1·01) vs. 0·75 (0·49 to 1·14)	0.9 (-1.5 to 3.1) vs. 2.2 (0.1 to 4.0) vs. 3.4 (-0.9 to 7.6)	8 RCTs [9, 10, 15, 16, 19–22]; 3,224 patients	No significant HTE; based on **post-hoc** subgroup analysis
	Time between hospitalization and treatment initiation	< 24 h vs. ≥ 24 h	0·30 (0·16 to 0·56) vs. 0·99 (0·52 to 2·01)*	9.8 (4.9 to 14.5) vs. 0.1 (-5.3 to 4.9)	1 RCT [10]; 794 patients	**Significant** HTE, suggesting more benefit from treatment within 24 h after hospitalization; based on **post-hoc** subgroup analysis

## Data Availability

No datasets were generated or analysed during the current study.

## Abbreviations

HTE: Heterogeneity of treatment effect
CAP: Community-acquired pneumonia
AD-MA: Aggregate-data meta-analysis
IPDMA: Individual patient data meta-analysis
CRP: C-reactive protein
PSI: Pneumonia severity index
